# High glucose mediates endothelial-to-chondrocyte transition in human aortic endothelial cells

**DOI:** 10.1186/1475-2840-11-113

**Published:** 2012-09-22

**Authors:** Rining Tang, Min Gao, Min Wu, Hong Liu, Xiaoliang Zhang, Bicheng Liu

**Affiliations:** 1 Institute of Nephrology, ZhongDa Hospital, school of medicine, Southeast University, 210009, Nanjing, China

**Keywords:** High glucose, Vascular calcification, Endothelial-to-mesenchymal transition, Mesenchymal stem cells, Snail

## Abstract

**Background:**

Vascular calcification is one of the common complications in diabetes mellitus. Many studies have shown that high glucose (HG) caused cardiovascular calcification, but its underlying mechanism is not fully understood. Recently, medial calcification has been most commonly described in the vessels of patients with diabetes. Chondrocytes were involved in the medial calcification. Recent studies have shown that the conversion into mesenchymal stem cells (MSCs) via the endothelial-to-mesenchymal transition (EndMT) could be triggered in chondrocytes. Our previous research has indicated that HG induced EndMT in human aortic endothelial cells (HAECs). Therefore, we addressed the question of whether HG-induced EndMT could be transitioned into MSCs and differentiated into chondrocytes.

**Methods:**

HAECs were divided into three groups: a normal glucose (NG) group, HG group (30 mmol/L), and mannitol (5.5 mmol/L NG + 24.5 mmol/L) group. Pathological changes were investigated using fluorescence microscopy and electron microscopy. Immunofluorescence staining was performed to detect the co-expression of endothelial markers, such as CD31, and fibroblast markers, such as fibroblast-specific protein 1 (FSP-1). The expression of FSP-1 was detected by real time-PCR and western blots. Endothelial-derived MSCs were grown in MSC medium for one week. The expression of the MSCs markers STRO-1, CD44, CD10 and the chondrocyte marker SOX9 was detected by immunofluorescence staining and western blots. Chondrocyte expression was detected by alcian blue staining. Calcium deposits were analyzed by alizarin red staining.

**Results:**

The incubation of HAECs exposed to HG resulted in a fibroblast-like phenotype. Double staining of the HAECs indicated a co-localization of CD31 and FSP-1. The expression of FSP-1 was significantly increased in the HG group, and the cells undergoing EndMT also expressed STRO-1, CD44 and SOX9 compared with the controls (*P* < 0.05). Additionally, alcian blue staining in the HG group was positive compared to the NG group. Consistent with the evaluation of SOX9 expression, calcium deposits analyzed by alizarin red staining were also enhanced by the HG treatment. Specifically, we showed that HG-induced EndMT is accompanied by the activation of the canonical Snail pathway.

**Conclusions:**

Our study demonstrated that HG could induce endothelial cells transdifferentiation into chondrocyte-like cells via the EndMT, which is mediated in part by the activation of the Snail signaling pathway.

## Background

Cardiovascular complications are the leading cause of death in patients with diabetes mellitus (DM) [1]. Emerging evidence suggests that the presence of vascular calcification in any arterial wall in patients with DM is associated with a 3-4-fold higher risk for mortality and cardiovascular events [2]. Pathological studies have shown that patients with DM exhibit characteristic calcification in the tunica media, which is independently associated with cardiovascular mortality [3]. However, the pathogenesis of medial artery calcification (MAC) is complex and has not been fully elucidated.

Traditionally, a calcium-phosphorus homeostasis imbalance is considered the cause of calcium salt deposition, which may play an important role in vascular calcification by transforming vascular smooth muscle cells (SMCs) to osteoblast-like cells, producing a matrix of bone collagen and non-collagenous proteins. MAC has recently been considered an orchestrated process that begins with mesenchymal condensation, followed by chondrogenesis and endochondral ossification [4,5]. However, the mechanisms responsible for these processes in diabetic vascular calcification remain largely unknown.

In the past decade, SMCs and pericytes have been reported to possess the plasticity to express bone and cartilage proteins in calcified blood vessels [6,7]. However, recent studies have indicated that mature endothelial cells can transform into fibroblasts in vitro and in DM [8-11], by a process known as the endothelial-mesenchymal transition (EndMT). Furthermore, Medici et al. found that vascular endothelial cells have the potential to convert into mesenchymal cells that possess mesenchymal stem cells (MSCs) properties and are able to differentiate into chondrocytes [12]. In addition, chondrocyte conversion underlies medial calcification in uremic rats [13]. Thus, whether EndMT might be a novel source of chondrocytes during the MAC process in patients with DM is an interesting question.

Chronic high glucose (HG) is a major initiator of diabetic vascular complications and implicated in the development of vascular calcification [14,15]. Our previous studies have shown that 30 mmol/L HG induced human aortic endothelial damage via the mediation of EndMT and that EndMT contributed to cardiac fibrosis in diabetic rats, which was inhibited by irbesartan [16,17]. However, the relationship between HG-induced EndMT and its association with chondrocyte transformation during diabetic vascular calcification are still poorly understood. In this study, we addressed the question of whether HG-induced EndMT could be used to transition human aortic endothelial cells (HAECs) into MSCs and then differentiate into chondrocytes.

## Materials and methods

### Cell culture

Primary HAECs were purchased from Sciencell Research Laboratories (USA) and cultured as previously described [16]. Briefly, cells were grown in endothelial culture medium (No. 1001, Sciencell) containing 5% fetal bovine serum (FBS) (No. 0025), 1% endothelial cell growth supplement (No. 1052) and 1% penicillin/streptomycin solution (No. 0503) in 5% CO_2_ at 37°C. Passage 2–5 HAECs were expanded in monolayers in flasks or dishes. At approximately 80% confluence, the culture medium was changed to a serum-free solution for 24 h prior to their use in all experiments. And the HAECs were treated with normal glucose (NG; 5.5 mmol/L), HG (30 mmol/L D-glucose) [16], or 5.5 mmol/L NG + 24.5 mmol/L mannitol for 48 h.

### Cell differentiation

The cells were grown in chondrogenic differentiation medium (MCDM, Sciencell) supplemented with TGF-β3 (Peprotech, Rocky Hill, USA) at a concentration of 10 ng/ml, followed by growth in serum-free medium with HG at 30 mmol/L for 48 h. Alcian blue (Sigma) staining for chondrogenic proteoglycans and alizarin red staining for calcium deposition were performed on cultures grown in chondrogenic medium for 7 days.

### Real-time PCR

The total RNA from the cultured HAECs was extracted using RNAiso Plus according to the manufacturer’s protocol (TAKARA, China). The RNA concentration and purity were confirmed with a Nanodrop 2000 (Thermo, USA). Samples with a relative absorbance ratio between 1.8 and 2.0 at 260/280 were used. All RNA samples were reverse transcribed (Applied Biosystems, USA).

The quantification of specific mRNAs was conducted using an ABI Prism 7300 Sequence Detection System (Applied Biosystems, USA) with the SYBR Green Real-time PCR Kit (TAKARA, China). The following oligonucleotide primer sequences were used: CD31, forward 5′-GAGTCCAGCCGCATATCC-3′ and reverse 5′-TGACACAATCGTATCTTCCTTC-3′; FSP1, forward 5′- GTCCACCTTCCACAAGTAC-3′ and reverse 5′ TGTCCAAGTTGCTCATCAG-3; CD44, forward 5′-GAGCAGCACTTCAGGAGGTTAC-3′ and reverse 5′-GGAATGTGTCTTGGTCTCTGGTAG-3′; CD10, forward 5′-CCTCGTTGACTGGTGGACTC-3′ and reverse 5′-CTGATAGGCTCTGTATGCTTGAC-3′; SOX9, forward 5′-GCTCTGGAGACTTCTGAAC-3′ and reverse 5′-CGTTCTTCACCGACTTCC-3′; and β-actin, forward 5′-CTGGAAGGTGGACAGCGAGG-3′ and reverse 5′-TGACGTGGACATCCGCAAAG-3′. All primers were designed and synthesized by Generay (Shanghai). The relative amount of mRNA was normalized to β-actin and calculated using the standard curve method. In brief, the pre-PCR product of each gene was used as the standard. The standard curve was established with a 10-fold serial dilution of the product and was included in all PCR runs. The ratio of target gene abundance to housekeeping gene abundance was used to evaluate the expression level of each gene. Controls consisting of ddH_2_O were negative in all runs.

### Western blot analysis

The total cellular protein was extracted to evaluate the levels of CD31, FSP1, α-SMA, CD44, STRO-1 and CD10. Equal amounts of cell lysate proteins (30 μg) were separated on 4-20% SDS-polyacrylamide gels and transferred onto nitrocellulose membranes (Pall, USA) by electroblotting. The blots were incubated overnight with primary antibodies at concentrations recommended by the respective manufacturers: CD31 (sc-65260, Santa Cruz), FSP1 (ab27957, Abcam), CD44 (sc-71220, Santa Cruz), and CD10 (sc-9149, Santa Cruz). A horse-radish peroxidase-labeled secondary IgG (Santa Cruz, Europe) was then added to the blots. The signals were detected using an ECL advance system (GE Healthcare, UK). β-actin was used as the internal control.

### Immunofluorescence

HAECs grown on coverslips were fixed in 4% paraformaldehyde and permeabilized with 0.3% Trition-X100. After blocking with 10% BSA for 1 h, they were incubated with primary antibodies at 4°C overnight. The cells were then incubated with AlexaFluor-conjugated secondary antibodies (Invitrogen Technology, USA) at room temperature in the dark for 1 h. The absence of primary antibody was used as a negative control. The cells were visualized and photographed with a scanning confocal microscope (LSM 510 META, Carl Zeiss, Germany; TCS SP5, Leica, Germany).

### Electron microscopy

Ultra-thin cells were counter-stained with uranyl acetate and lead citrate and were examined with a transmission electron microscope (TEM, HITACHI H600). Dried samples were sputtered with gold for observation by a scanning electron microscope (SEM-505, Phillips, the Netherlands).

### Statistical analysis

The data were expressed as the means ± standard deviation (SD). A one-way analysis of variance (ANOVA) was performed and confirmed with a two-tailed paired Student’s *t* test using SPSS 19.0. *P* values less than 0.5 were considered significant.

## Results

### HG induces EndMT in HAECs

**Figure 1:**
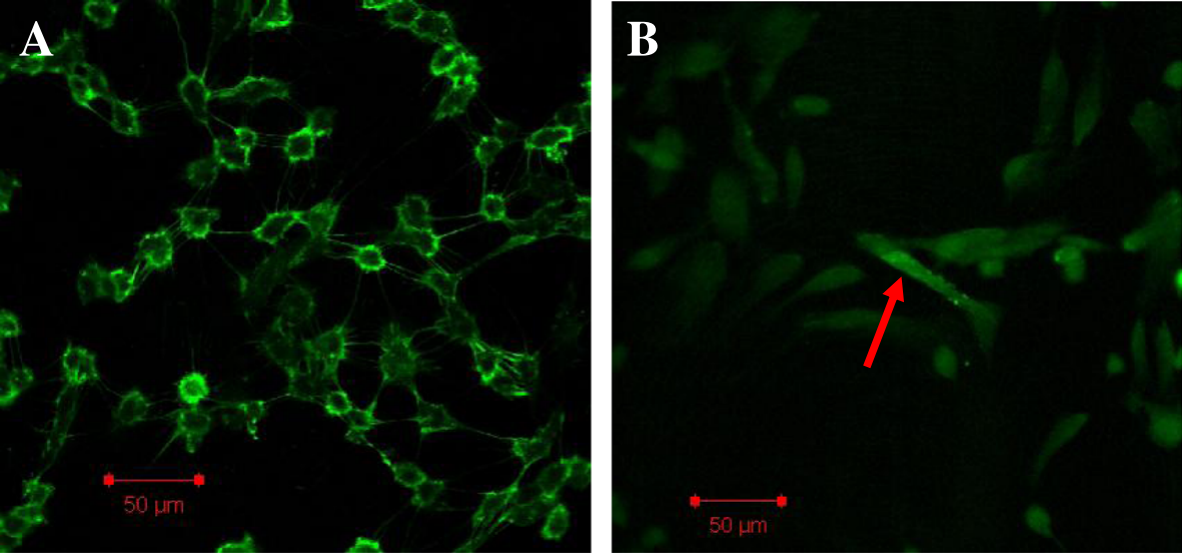
**Immunofluorescence staining of HAECs with CD31 in the NG and HG groups.** Note: The incubation of HAECs with high glucose (30 mmol/L) for 48 h resulted in a fibroblast-like phenotype (**B**). 1 bar = 50 μm. **A**: normal glucose. **B**: high glucose.

**Figure 2:**
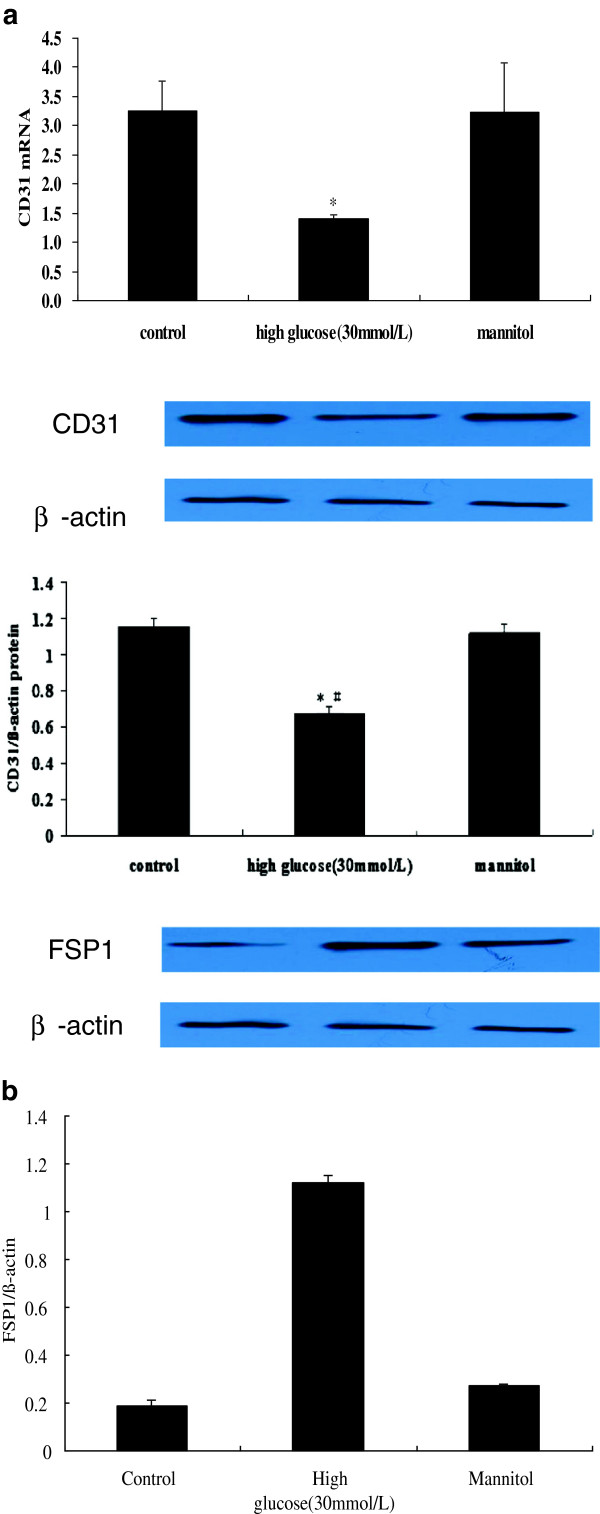
**RT-PCR and western blot expression of CD31 in the NG and HG groups.** Notes: The mRNA and protein expression of CD31 were decreased in cells incubated with high glucose, whereas the expression of FSP-1 was increased. * *P* < 0.05 *vs.* control; ^#^*P* < 0.05 *vs.* mannitol.

**Figure 3:**
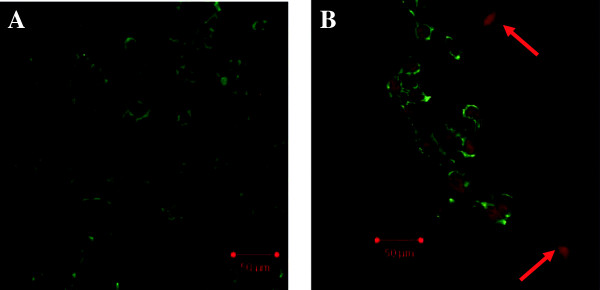
**The confocal microscopy analysis.** Notes: Labeling experiments used antibodies against CD31 (endothelial cell marker; green) and the fibroblast marker FSP1 (red). Confocal microscopy revealed that some cells acquired FSP1 staining and lost CD31 staining, which suggested the stage of EndMT. **A**: normal glucose. **B**: high glucose.

As our previous experiment showed, we found that HAECs treated with 30 mmol/L HG for 48 h induced profound changes, with the cells becoming elongated, spindle-shaped and losing cobblestone morphology under fluorescence microscopy (Figure 1). The protein and mRNA expressions of endothelial marker CD31 were decreased in the cells incubated with HG, whereas the protein expression of FSP-1 was increased (Figure 2). Immunofluorescence with antibodies for the endothelial marker and the mesenchymal marker demonstrated that the HG-treated cells acquired FSP1 staining and lost CD31 staining compared with the control cells (Figure 3).

**Figure 4:**
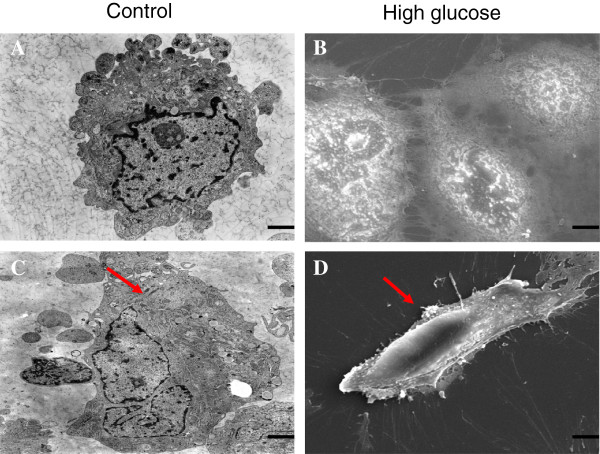
**Cellular ultrastructure following HG treatment.** Notes: TEM and SEM depicting the change in the cellular ultrastructure following high glucose (30 mmol/L) exposure. The normal HAECs present with few microfilaments and a rough endoplasmic reticulum (**A**, **B**). After exposure to HG, microfilamentation and a swollen rough endoplasmic reticulum appeared in the cytoplasm, and the cells became elongated and spindle-shaped (**C**, **D**). 1 bar = 4 μm.

We next observed the cells under TEM and SEM. HAECs treated with 30 mmol/L HG for 48 h showed a distinct change from a cobblestone-like to a spindle-shaped morphology. In addition, TEM was performed to examine the ultrastructure of cells, which displayed normal structures. In contrast, the HG group treated for 48 h exhibited endothelial protrusion, a significantly roughened endoplasmic reticulum, and microfilamentation (Figure 4). These data suggested that EndMT was induced by the HG treatment.

### HG-induced EndMT expresses mesenchymal stem markers and acquires chondrocyte differentiation potential

**Figure 5:**
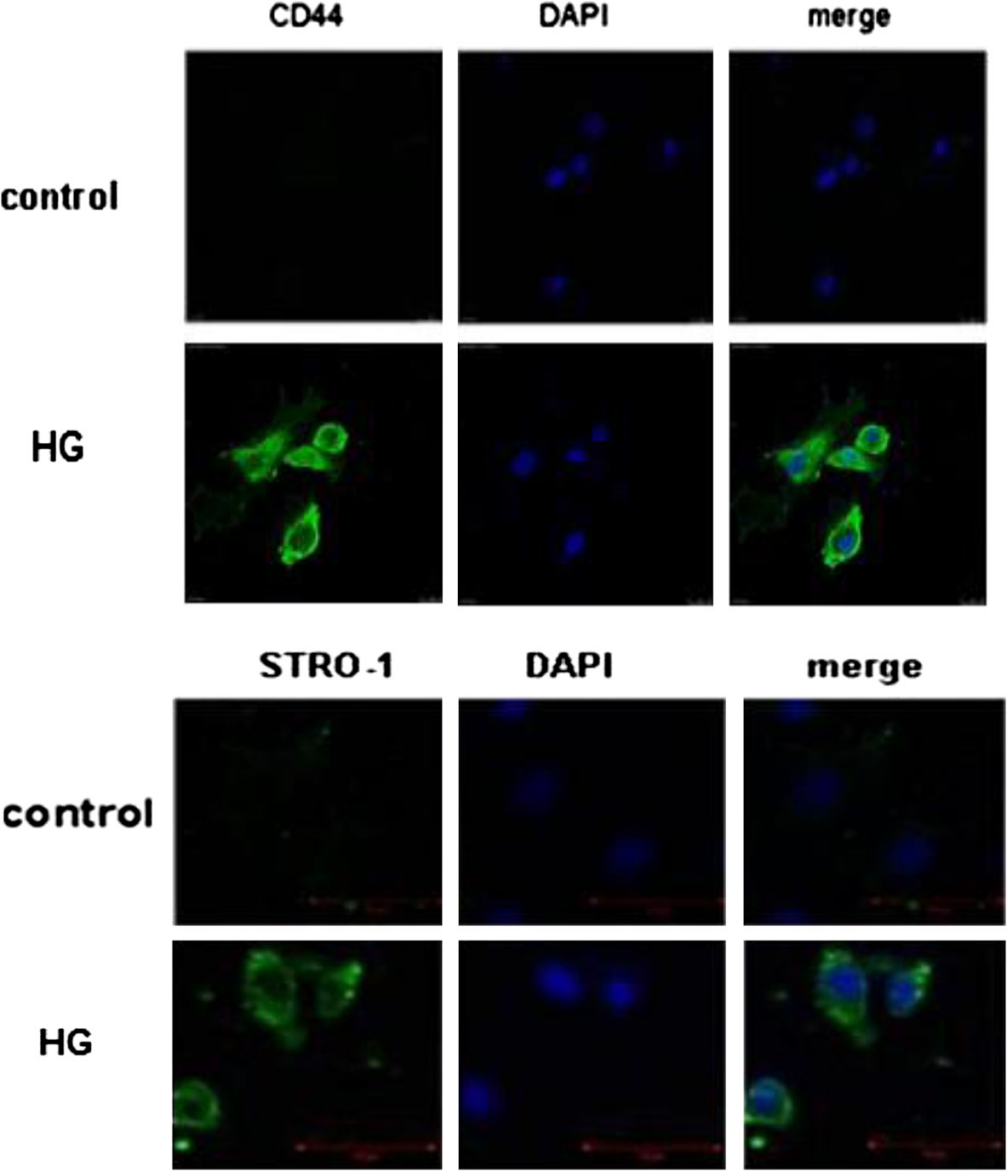
**Immunofluorescence staining of HAECs with CD44 and STRO-1 in the NG and HG groups.** Note: Representative immunofluorescence staining of CD44 and STRO-1 (green) with DAPI. 1 bar = 50 μm. Control: normal glucose. HG: high glucose.

**Figure 6:**
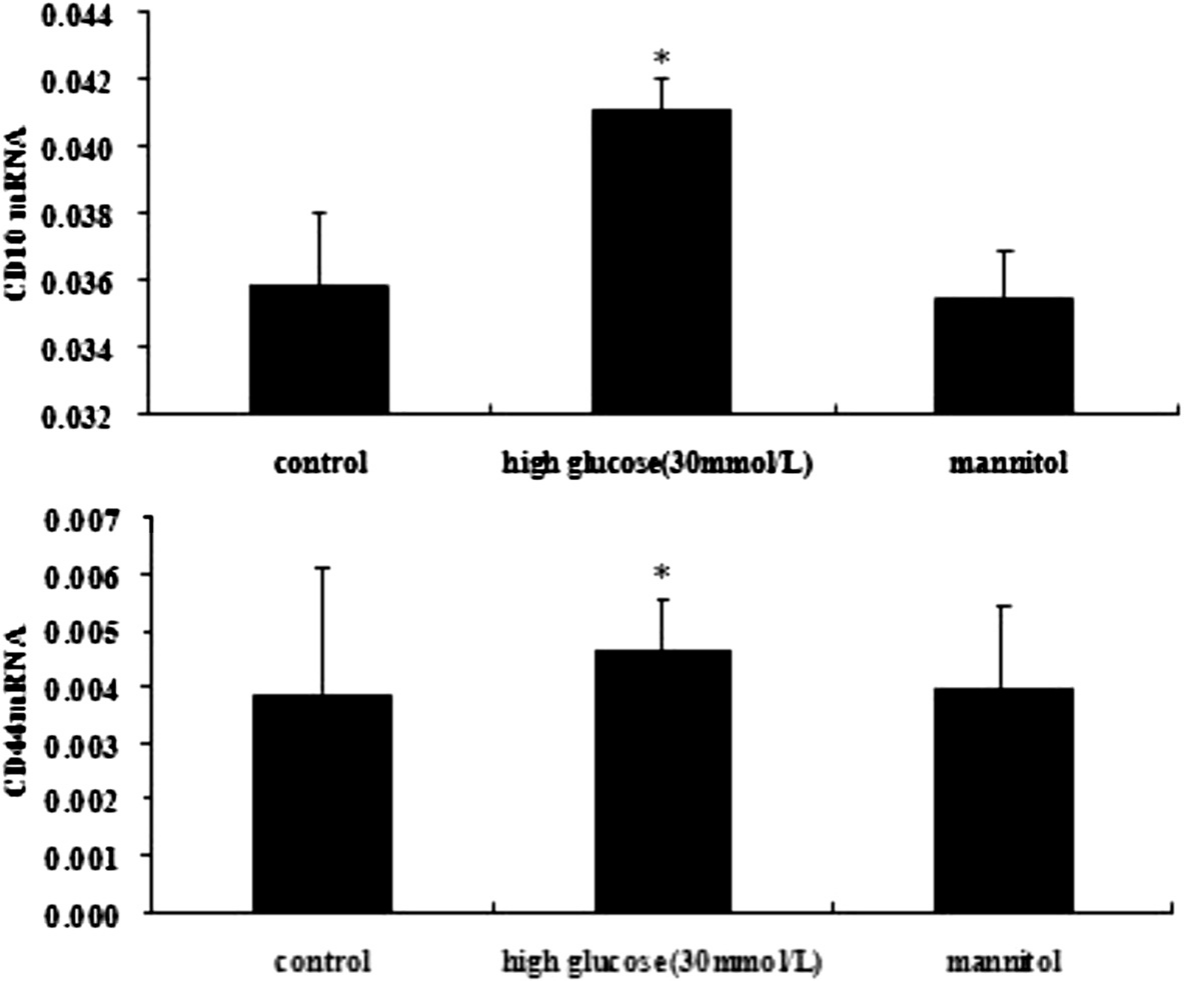
**RT-PCR expression of CD10 and CD44 in the NG and HG groups.** Notes: HAECs were treated with 30 mmol/L high glucose for the indicated times. The expression of MSCs markers (CD44, CD10) was detected by real-time PCR. The values represent the means ± SD. *, *P* < 0.05 *vs.* control.

**Figure 7:**
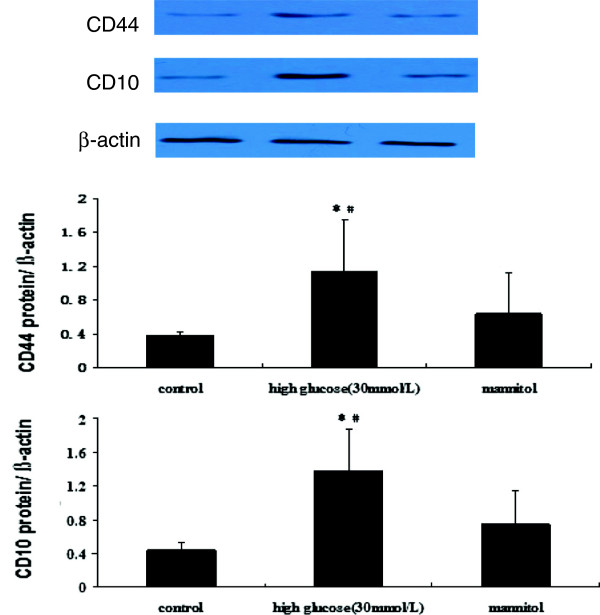
**Western blot expression of CD44 and CD10 in the NG and HG groups.** Notes: HAECs were treated with or without 30 mmol/L HG for 48 h. MSCs marker (CD44, CD10) expressions were detected by western blot. β-actin was used as internal control. Values represent the means ± SD. *P < 0.05 *vs.* control. ^#^*P* < 0.05 *vs.* mannitol.

To determine whether EndMT could cause an acquisition of MSCs-like phenotype, HAECs were treated with HG to examine the expression of the MSC markers CD44, CD10 and STRO-1. We performed immunofluorescence with antibodies for the MSC markers CD44 and STRO-1. After incubation with 30 mmol/L HG for 48 h, the cells showed significantly increased expression of CD44 and STRO-1 compared with the control under confocal microscopy (Figure 5). Compared with the control group, the HG treatment induced significantly increased mRNA expression in CD44 and CD10 at 48 h post-treatment (Figure 6). In addition, the CD44 and CD10 protein expression was markedly increased (Figure 7).

**Figure 8:**
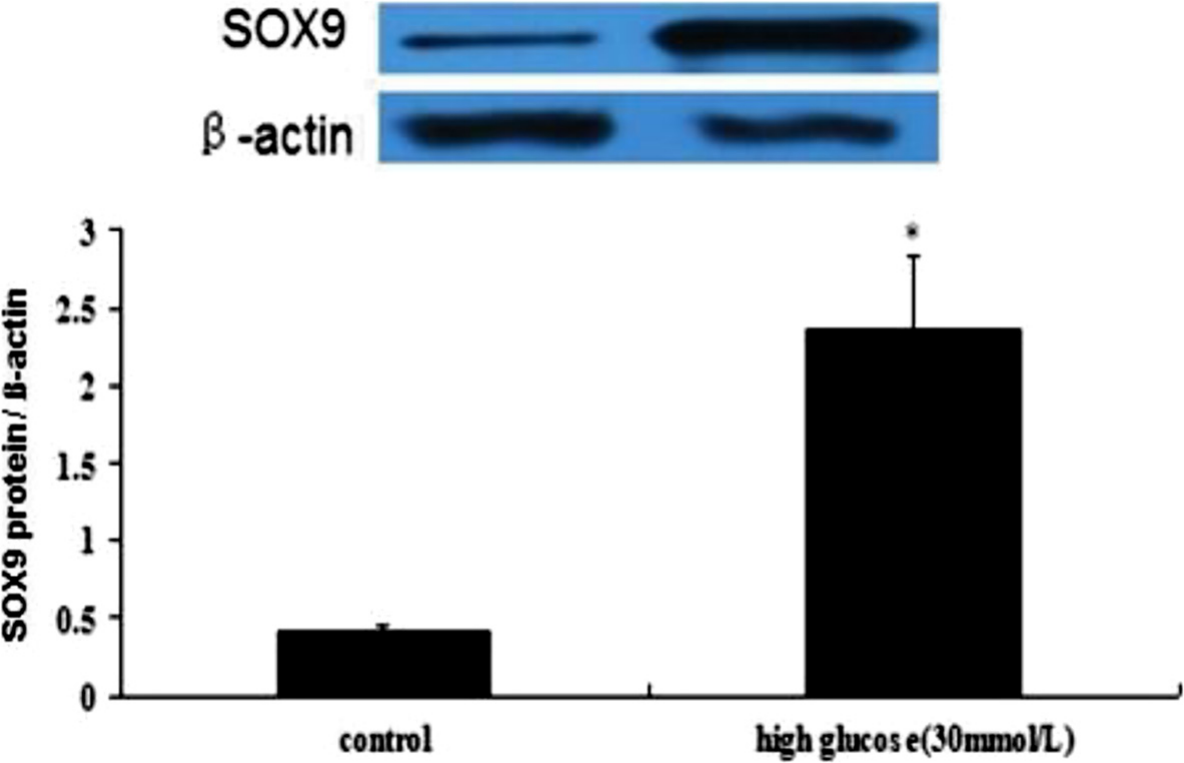
**Western blot expression of SOX9 in the NG and HG groups.** Notes: Western blot analysis for the chondrocyte marker is shown (SOX9). β-actin was used as an internal control. The values represent the means ± SD. *, *P* < 0.05 *vs.* control.

**Figure 9:**
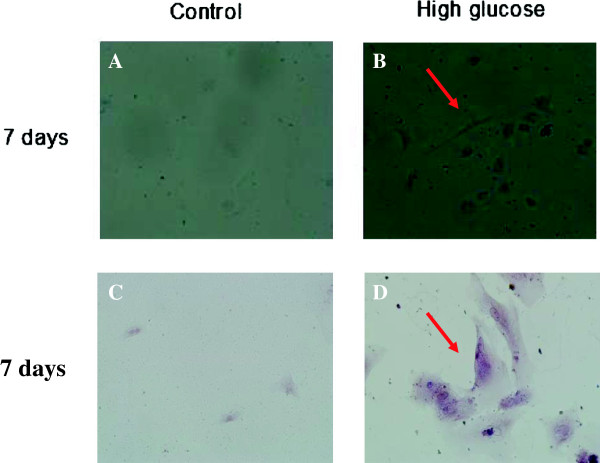
**HAECs incubated with HG exhibit chondrocyte differentiation potency.** Notes: HAECs were treated with or without 30 mM HG for 48 h followed by exposure to chondrogenic culture medium for 7 days (**A**). Alcian blue staining for cells grown in chondrogenic culture medium for 7 days is shown (**B**, ×100). (**C**). Alizarin Red staining for calcium deposition (**D**, ×200).

Because MSCs are multipotent, we next assessed the chondrocyte differentiation capability of endothelial cells that underwent EndMT. We exposed the ECs to the 30 mmol/L HG for 48 h after the cells were grown in chondrogenic culture media for one week. Western blot analysis showed that the expression of the chondrocyte-specific marker SOX9 was significantly increased compared with the control (Figure 8). Meanwhile, the HG-treated HAECs stained positively for cartilage proteoglycan alcian blue after growth in chondrogenic medium for 7 days (Figure 9). Consistent with the evaluation of SOX9 expression, calcium deposits as shown by alizarin red staining were also enhanced by HG treatment (Figure 9).

### HG-induced EndMT is associated with the activation of the Snail signaling pathway

**Figure 10:**
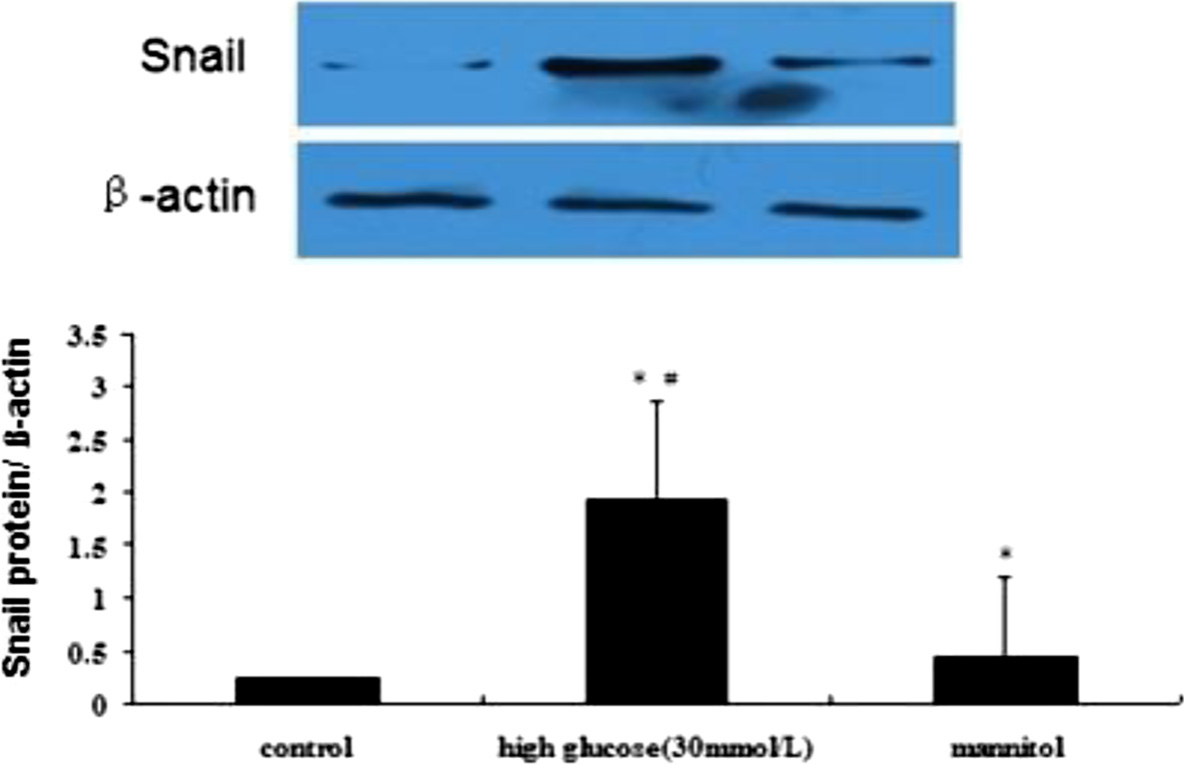
**Western blot expression of Snail in the NG and HG groups.** Notes: HAECs were treated with 30 mmol/L high glucose for 48 h followed by exposure to chondrogenic culture medium for 7 days. The western blot analysis for Snail is shown. β-actin was used as an internal control. The values represent the means ± SD. *, *P* < 0.05 *vs.* control.

To determine whether the EndMT triggered by HG observed in our experiments was accompanied by the activation of Snail, a western blot was performed using HAECs treated with or without HG. The protein level of Snail was significantly increased by treatment with HG compared with the control (Figure 10).

## Discussion

Vascular calcification is more common in patients with diabetes compared with the general population and is associated with increased mortality, stroke and amputations [1-3]. In addition, under HG conditions with increased TNF-alpha levels, the death receptors TNF-R1 and Fas, are up-regulated in human coronary artery endothelial cells, which could in turn play a role in HG-induced endothelial cell apoptosis and hyperglycemia appears to be a risk factor for vascular calcification [14-16]. Many studies have shown that HG may cause cardiovascular medial calcification and increased levels of plasma Matrix GLA protein indicates the progressing calcification process in patients with type 2 DM [17], but its underlying mechanism is not fully understood in HG-induced endothelial injury. We repeated the same results as a previous study: it showed that the treatment of HAECs with HG led to a significantly increased expression of the FSP1 protein in a dose- and time-dependent manner (data not shown). Double staining of the HAECs showed co-localization of CD31 and FSP1, the acquisition of a spindle-shaped morphology by some cells and loss of CD31 staining, which indicated the occurrence of EndMT [18,19]. Furthermore, in this study, the cells undergoing EndMT showed expression of STRO-1, CD44 and SOX9 compared with the controls. Additionally, alcian blue staining in the HG group was positive compared with the NG group. The EndMT resulted in the acquisition of MSCs-like properties to enable the differentiation into chondrocytes, which is in accordance with the report from Medici and colleagues [12]. We first found that the HG-induced EndMT could trigger the conversion into chondrocytes, which are involved in the vascular medial calcification.

Chondrogenesis is a key pathologic mechanism underlying medial calcification [4,13]. Given its central role, it is not surprising that the origins of ectopically accumulated chondrocytes in the arterial wall have become an attractive field in research. During the past decade, the notion that SMCs undergo differentiation into chondrogenic cells to participate in MAC has already gained acceptance [5,7]. However, conventional distinctions among vascular cell lineages are becoming less clear as our studies and other investigators determine that ECs could also trans-differentiate into SMCs, a process known as EndMT [10,11,18,19]. Furthermore, studies found that EndMT has the ability to convert cells into MSCs and then differentiate into chondrocytes [12].

MSCs are known to be multipotent cells with cartilage-, adipose-, and bone-forming potential that are widespread in calcified lesions [20-23]. Previous studies have determined that MSCs in adult humans and rodents are derived from bone marrow, cord blood, placenta and adipose tissue [21,23]. Recently, vascular endothelial cells have been proposed to be one precursor of MSCs in certain microenvironments [21,24]. TGF-β2 treatment-induced EndMT contributes to the acquisition of the MSC phenotype in ECs [12,23]. Kissa et al. [25] indicated that ECs from the aortic ventral wall can transformation into hematopoietic cells in animals. Slukvin et al. [24] showed the MSCs could derive from EndMT. More interestingly, Medici et al. found that chondrocytes at the sites of heterotopic ossification stain positively with antibodies specific for endothelial markers in progressive models of fibrodysplasia ossificans [12]. In our experiment, we found that elevated HG could induce the significantly increased expression of CD44, CD10, and STRO-1 (markers of MSCs) and SOX9 (a transcription factor required for chondrocyte differentiation) in cells undergoing EndMT. Meanwhile, HG-treated HAECs stained positively for cartilage proteoglycan alcian blue after growth in chondrogenic medium for 7 days. Consistent with the evaluation of SOX9 expression, calcium deposits as shown by alizarin red staining were also enhanced by HG treatment. All of these experiments suggested that the endothelium may contribute to chondrocyte genesis by MSCs generation, which was involved in the diabetic vascular medial calcification.

Recent work has shown that the exposure of ECs to HG activates several signal transduction networks responsible for mediating the proliferative and growth-promoting responses. Snail has been implicated in a wide variety of cellular responses, including transition, growth, gene expression, angiogenesis, contractility and vesicle trafficking. Kokudo et al. [26] found that Snail is required for the TGF ß-induced EndMT of embryonic stem cell-derived ECs. Moreover, TGF-β2 promotes Snail-mediated endothelial-mesenchymal transition through the convergence of Smad-dependent and Smad-independent signaling [27]. In addition, a role of the TGF-β in the regulation of terminal chondrocyte differentiation was recently reported [28]. Jayachandran and colleagues [29] found that Snail transcription factors mediate the epithelial-mesenchymal transition (EMT) in lung fibrosis. EndMT is known to be a form of EMT that is present during the embryonic development of the heart [30], and glycogen synthase kinase-3 is an endogenous inhibitor of Snail transcription, which has been implicated in EMT [31]. Thus, we addressed the question of whether Snail mediated the HG-induced EndMT. Our results indicated that the protein level of Snail was significantly increased by the HG treatment compared with the control. Therefore, all of these findings support the hypothesis that the effect of HG on EC transdifferentiation into chondrocyte-like cells is mediated, at least in part, through the Snail signaling pathway.

## Conclusions

Our study demonstrated that HG could induce endothelial cells transdifferentiation into chondrocyte-like cells via EndMT, which is mediated in part by the activation of the snail signaling pathway. Understanding the mechanisms that control endothelium transdifferentiation to osteochondroprogenitors and the subsequent vascular calcification may help develop novel strategies that prevent or reverse vascular calcification.

## Abbreviations

EndMT: Endothelial-to-mesenchymal transition; DM: Diabetes mellitus; FSP-1: Fibroblast-specific protein1; HAECs: Human aortic endothelial cells; HG: High glucose; MSCs: Mesenchymal stem cells; MA: Cmedial artery calcification; NG: Normal glucose; SMCs: Smooth muscle cells.

## Competing interests

The authors declare that they have no competing interests.

## Authors’ contributions

TR: conception and design, data interpretation, manuscript writing and final approval of manuscript; WM: conception and design, data analysis, manuscript writing, final approval of manuscript; LH and GM: advice with protein assay and data interpretation; LB: conception and design, assembly of data and data interpretation, manuscript review, final approval of manuscript. All authors read and approved the final manuscript.

## Authors’ information

Min Gao as Co-first author.

Min Wu as Co-corresponding author.
